# Phalloplasty Following Penectomy for Fournier’s Gangrene at a Tertiary Care Center

**DOI:** 10.7759/cureus.3698

**Published:** 2018-12-06

**Authors:** Don Hoang, Pedram Goel, Vivi W Chen, Joseph Carey

**Affiliations:** 1 Plastic Surgery, University of Southern California Keck School of Medicine, Los Angeles, USA

**Keywords:** phalloplasty, penectomy, reconstruction, fournier’s gangrene, dermal regeneration template, free flap

## Abstract

Treatment of Fournier’s gangrene often requires extensive surgical debridements that can ultimately necessitate penile amputation. Reconstruction can be challenging as these patients tend to have medical comorbidities deeming them poor microsurgical candidates. Fournier’s gangrene resulting in penectomy is an infrequent occurrence, and treatment with phalloplasty is rarely described in the literature. Herein, we present a case of a 60-year-old male with poorly controlled diabetes mellitus who developed Fournier’s gangrene in July 2017. His treatment course included multiple surgical debridements without resolution, eventually necessitating a penectomy. The patient elected for surgical reconstruction and underwent a phalloplasty procedure utilizing a radial forearm free flap. This case demonstrates a rare case of Fournier’s gangrene resulting in penectomy with a unique reconstruction utilizing a radial forearm free flap in a poor microsurgical candidate.

## Introduction

Fournier’s gangrene is a soft tissue polymicrobial infection involving the genitals and perineum. These infections commonly affect patients with longstanding poorly controlled diabetes and vascular disease. Although Fournier’s gangrene typically involves the genitals, infections limited to the penis are rare due to its rich vascular supply [1]. Further, treating these infections with total penectomy are seldom necessary [2].

Should a patient require a penectomy to prevent extension of infection to more vital parts of the body, the reconstructive options allowing return of form and function may be limited. One solution involves free tissue transfers from healthy regions of the body to reconstruct the phallus. In fact, this phalloplasty procedure has seen a recent upsurge secondary to gender reassignment surgery [3]. Although phalloplasty in a young, healthy patient undergoing gender reassignment surgery can have satisfactory results, phalloplasty for the treatment of Fournier’s gangrene has substantial risk due to the patient’s likely comorbidities and vascular disease, making these patients poor microsurgical candidates with a great risk of flap failure. Preoperative blood sugar control and rigorous surgical preparation are therefore imperative in mitigating the risk of flap failure, while stringent surgical flap selection helps ensure the highest chance of viability.

There is a paucity of literature describing phalloplasty as a repair for penile loss secondary to Fournier’s gangrene. One study conducted at the University Teaching Hospital in Sokoto, Nigeria between 1994 and 2003 reported three phalloplasty procedures for treatment, without a description of the phalloplasty method or outcomes [4]. Another single-center study of 10 patients undergoing phallic reconstruction with a radial forearm free flap reported one patient who suffered penile loss secondary to Fournier’s gangrene but did not specify this specific patient’s outcomes [5]. Therefore, the aim of this study was to describe a unique case of Fournier’s gangrene isolated to the phallus requiring penectomy and utility of the radial forearm free flap in penile reconstruction following amputation.

## Case presentation

**Figure 1:**
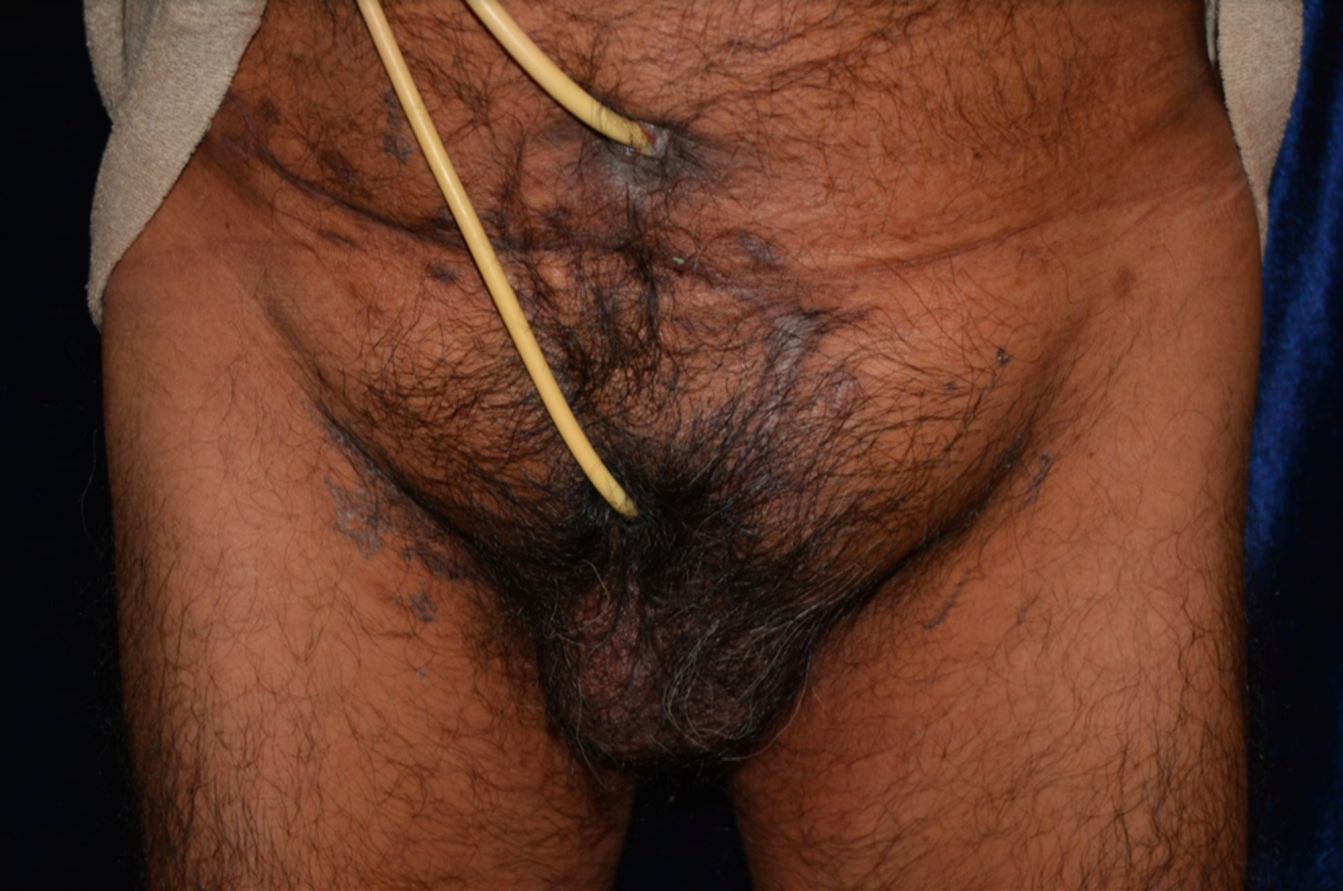
Preoperative view of a patient after penectomy with a Foley catheter in native urethra and a suprapubic catheter

This case involves a 60-year-old male with a history significant for benign prostatic hyperplasia, hypertension, and insulin-dependent diabetes who presented in July 2017 with diabetic ketoacidosis and Fournier’s gangrene. The patient’s treatment course for Fournier’s gangrene subsequently consisted of multiple operative debridements, an intensive care unit (ICU) admission, and ultimately a penectomy in July 2017 (Figure 1). He continued his post-discharge follow-up at an outside hospital for recuperative care as he became homeless during this time. He received wound care with home healthcare and continued to undergo a follow-up in the outpatient clinic at the urology and plastic and reconstructive surgery departments, where he discussed his desire to undergo penile reconstruction. After discussing all treatment options as well as the risks and benefits of the surgery, the patient agreed to move forward with surgical reconstruction. The patient obtained consent for a free flap procedure, and plans were discussed to proceed with a radial forearm reconstruction of his penis.

This procedure involved phallus and neourethra construction utilizing a single radial forearm free flap. First, a 12-cm circumferential circle was marked and dissected out at the planned penis insertion site. Next, two branches of the dorsal penile nerve were carefully isolated and dissected out along with the left deep inferior epigastric and vena comitans that provided approximately 8 cm of pedicle length. An 8-cm segment of the great saphenous vein was also dissected out and transposed to the inferior epigastric vessels to assist with the flap anastomosis. The recipient vessels and nerves were now defined and attention was turned to harvesting the radial forearm free flap. The radial forearm free flap was lifted in the subfascial plane, while special attention was given to preserving the cephalic vein, the basilic vein, and the radial sensory nerve. The medial and lateral antebrachial cutaneous nerves were then isolated, and the flap was fashioned into a neophallus and urethra prior to vessel and nerve ligation. Once completed, the nerves and vessels were ligated, the flap was transferred to the groin for microsurgery, and the radial forearm donor site was covered with Integra (Integra LifeSciences, Plainsboro, NJ). The radial artery was anastomosed with the left deep inferior epigastric artery, two vena comitans were coupled to the greater saphenous vein, and two nerves were coapted to the dorsal penile nerve stump. The procedure was concluded and the patient was transferred to the ICU in stable condition. Postoperatively, the patient developed a 1 x 1.5-cm^2^ area of distal flap tip necrosis that resolved following conservative treatment with daily topical Silvadene. The patients remained in the ICU postoperatively for close flap monitoring and nutritional optimization, and he was discharged 14 days following the procedure. The postoperative course was complicated by a flap and donor-site infection requiring debridement of both sites with subsequent resolution of the infection. The patient is pending anastomosis of his neourethra to his native urethral stump by the urology department. The patient currently has a catheter in place for a urinary function that will be removed only after the neourethral anastomosis is complete. Should the patient desire the ability to maintain an erection for sexual activity, he would require an additional surgery for penile prosthesis placement. The patient has reported preserved sensation in the phallus and expressed satisfaction with his result.

## Discussion

The phalloplasty procedure is often the surgery of choice for phallus reconstruction due to its superior cosmetic and functional results [6]. The goal of this surgery should be to conduct a one-step procedure that results in an aesthetically natural-appearing penis that is sensate and enables the patient to void and take part in sexual intercourse [7-8]. The radial forearm free flap is the most commonly utilized flap for this procedure as it allows surgeons to address these various goals, permitting patients to regain both sexual and urinary functions. This method allows surgeons to produce a relatively normal-appearing phallus with an incorporated neourethra that is at a lower risk for serious complications when compared with other techniques [9-10].

Phalloplasty secondary to penile loss as a result of Fournier’s gangrene is rare, with our search of literature yielding only two studies with four previous cases described [4-5]. Additionally, only one of these previous cases described the use of the radial forearm free flap for phallic reconstruction. To the best of our knowledge, no other cases of Fournier’s gangrene requiring penectomy for a definitive treatment followed by phalloplasty utilizing a radial forearm free flap have been described. Our patient’s case was unique because he would not traditionally be considered for microsurgical interventions. Recent developments in technology and surgical techniques have advanced the field of reconstructive microsurgery resulting in improved patient outcomes and complication rates over the past few decades [11-12]. These advancements combined with rigorous pre-operative planning and patient optimization allowed us to ensure successful reconstruction in this patient. Although Fournier’s gangrene resulting in isolated penectomy is rare, free flap reconstruction should be carefully considered as a viable option in select patients for reconstruction.

**Figure 2:**
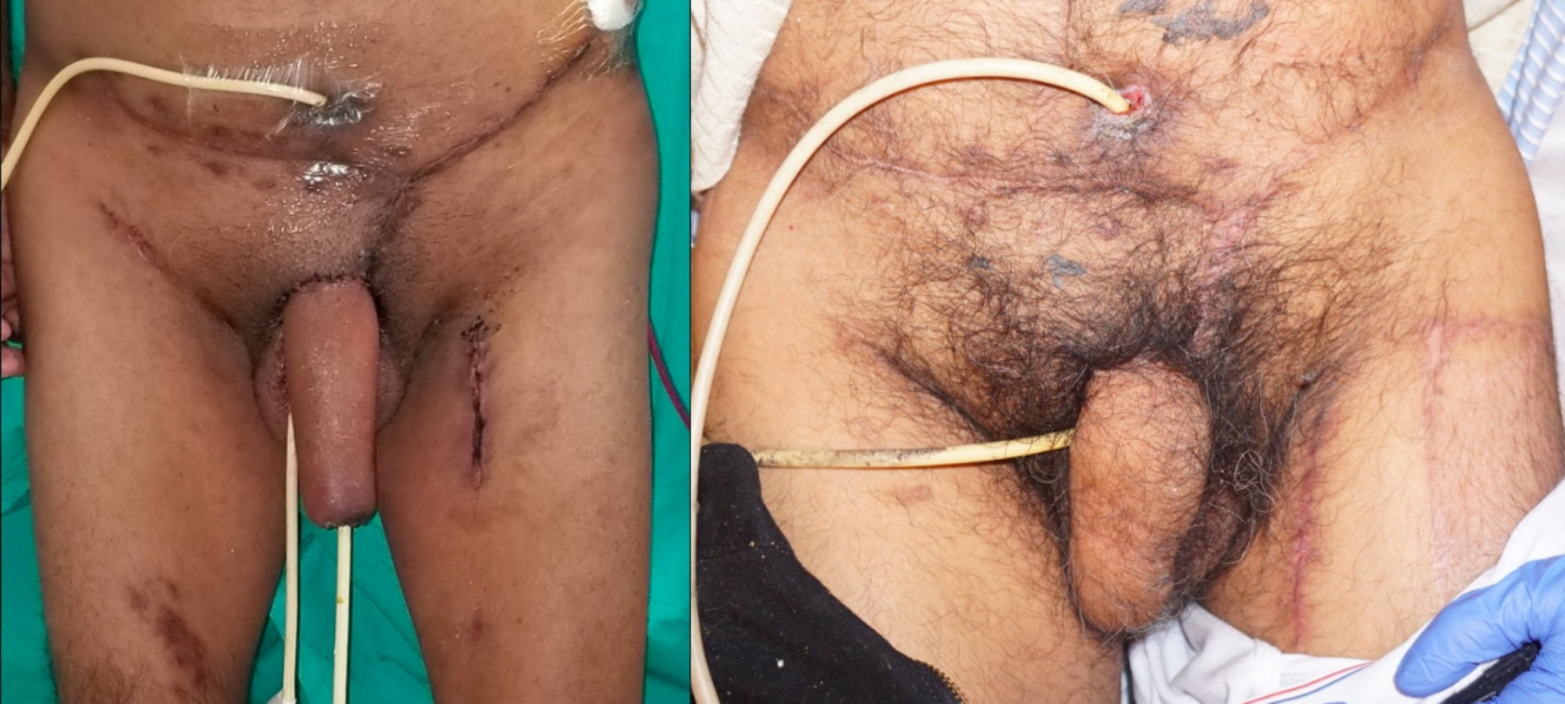
Postoperative view of patient after phalloplasty at two weeks (left) and at two months (right)

During pre-operative planning, it was imperative that our approach minimizes the risk for infections and complications, as these risks are amplified in patients with comorbid poorly controlled diabetes mellitus. We, therefore, determined that the radial forearm free flap was the most appropriate method to reduce these risks and allow us to complete the phalloplasty in a single procedure. Special care must be taken during surgical planning of the neourethral position in the free flap as only relatively hairless skin should be used to prevent stone formation and recurrent urinary tract infections [5]. This method allowed us to meet our pre-operative goal involving creating a neophallus and neourethra that was sensate at two months postoperatively in a single operation (Figure 2). While favorable results were achieved in our patient, this method of reconstruction is limited by the major donor-site morbidity that may require full- or split-thickness skin grafting or other dermal regeneration products as well as the need for microsurgical techniques, which increase the risk of failure [10,13-14].

Various other surgical options are available for surgeons when considering a phalloplasty procedure each with their own benefits and limitations. An additional well-studied flap that can be utilized during a phalloplasty procedure incorporates the anterolateral thigh (ALT) flap. This flap is appropriate for patients who would like to prevent a visible scar on their forearm and instead prefer an easily hidden donor site with no requirement for microvascular anastomosis [3,15]. Additionally, the ALT flap can also be utilized in patients in whom the radial forearm free flap is not a viable option either due to vascular or anatomic anomalies or secondary to previous forearm surgery [15]. The disadvantages of this flap include a higher incidence of flap and urethral complications when compared to the radial forearm free flap and the need for multiple stages and revisions [15]. These previously stated factors made this procedure unsuitable for our medically complex patient who would be unlikely to tolerate multiple procedures and is already at an increased risk of complications. Finally, a latissimus dorsi flap can be used for phalloplasty, but the results may be limited by the lack of a sensory nerve in the donor flap and the potential bulk of the neophallus [14]. Each surgical technique has advantages and disadvantages that must be thoroughly considered by the surgeon to select the optimal method for reconstruction. Surgeons should discuss each option with their patients and make an effort to select a method that is best aligned with their reconstructive goals [9,14].

Traditionally, penile reconstruction has been extraordinarily complex, due to the difficulty in finding suitable replacements for erectile and urethral tissues [10]. In fact, the creation of a fully functional phallus remains elusive. However, according to a review of phalloplasty techniques and outcomes, techniques continue to evolve and have high reported satisfaction rates [9]. As previously mentioned, the radial forearm free flap is most common and is what was used in this patient that successfully allowed us to achieve our goals of reconstructing a sensate phallus in a single procedure. This case demonstrates that the radial forearm free flap’s utility should not be overlooked, even in patients who would not otherwise be considered as optimal microsurgical candidates, and therefore provides an important contribution to the existing literature by expanding the microsurgical boundaries. Ultimately, it will be important to conduct controlled studies to objectively assess the optimal techniques for patients who require this operation. Successful surgery can allow a patient to regain significant functional ability and quality of life, and it will be important to also consider that a surgical approach varies based on the individual patient. 

## Conclusions

Whether acquired or congenital, malformation or absence of the penis can cause severe psychological stress for the male patient. Successful penile reconstruction not only has the potential to alleviate the psychological burden associated with such a traumatic experience but also makes it possible for the patient to resume sexual activity. Fournier’s patients are at a significant risk of flap failure due to comorbidities, but this case demonstrates that even at an institution that serves patients with multiple comorbidities and complex social situations, with proper preparation and optimization, phalloplasty can be a viable and successful surgical option to help restore the patient’s quality of life.
